# Neonicotinoids target distinct nicotinic acetylcholine receptors and neurons, leading to differential risks to bumblebees

**DOI:** 10.1038/srep24764

**Published:** 2016-04-28

**Authors:** Christopher Moffat, Stephen T. Buckland, Andrew J. Samson, Robin McArthur, Victor Chamosa Pino, Karen A. Bollan, Jeffrey T.-J. Huang, Christopher N. Connolly

**Affiliations:** 1 Centre for Environmental Change and Human Resilience, University of Dundee, Dundee, DD1 9SY; 2 Centre for Research into Ecological and Environmental Modelling, University of St.Andrews, KY16 9LZ; 3 Biomarker and Drug Analysis Core Facility, School of Medicine, University of Dundee, Dundee, DD1 9SY.

## Abstract

There is growing concern over the risk to bee populations from neonicotinoid insecticides and the long-term consequences of reduced numbers of insect pollinators to essential ecosystem services and food security. Our knowledge of the risk of neonicotinoids to bees is based on studies of imidacloprid and thiamethoxam and these findings are extrapolated to clothianidin based on its higher potency at nicotinic acetylcholine receptors. This study addresses the specificity and consequences of all three neonicotinoids to determine their relative risk to bumblebees at field-relevant levels (2.5 ppb). We find compound-specific effects at all levels (individual cells, bees and whole colonies in semi-field conditions). Imidacloprid and clothianidin display distinct, overlapping, abilities to stimulate Kenyon cells, indicating the potential to differentially influence bumblebee behavior. Bee immobility was induced only by imidacloprid, and an increased vulnerability to clothianidin toxicity only occurred following chronic exposure to clothianidin or thiamethoxam. At the whole colony level, only thiamethoxam altered the sex ratio (more males present) and only clothianidin increased queen production. Finally, both imidacloprid and thiamethoxam caused deficits in colony strength, while no detrimental effects of clothianidin were observed. Given these findings, neonicotinoid risk needs to be considered independently for each compound and target species.

Insect pollinators are essential pollinators of many native wildflowers and agriculturally important crops. Although pesticides are widely used to protect our crops, they are also exposed to many beneficial insects and may be contributing to the worldwide decline in pollinators and other beneficial insects^1^,^2^. The economic value of insect pollination is around US$215 billion (2005)^3^, as pollination improves crop yield, quality, shelf life and commercial value^4^,^5^,^6^. The neonicotinoids are a widely used class of insecticides that are nicotine-based compounds often used as systemic insecticides (seed coating) on bee-visited crops such as oilseed rape (canola) and ornamental garden plants or as foliar sprays on top fruit such as apples and pears. Once absorbed into the plant following seed germination, neonicotinoids can translocate throughout the plant, contaminating (at low ppb) their nectar and pollen during crop flowering^7^. The crop nectar and pollen may be consumed by bees, or transported back to the nest to feed their larvae or for long-term storage. As neonicotinoids persist in the soil^8^, they may also compromise the function of soil organisms that contribute to soil fertility and so limit crop yield^9^. Once in the soil they can translocate to wildflowers in field margins^10^,^11^ and so prolong exposure to insect pollinators, well beyond crop flowering^12^ and even into the following year^8^.

Neonicotinoids act as nicotinic acetylcholine receptor (nAChR) agonists, which provide the majority of the excitatory neurotransmission in the insect central nervous system. Imidacloprid (IMD) is a partial agonist^13^, evoking 10–14% of maximal ACh-induced responses^14^ in Kenyon cells. However, thiamethoxam (THX) has been found to be an inactive pro-pesticide that lacks functional activity on isolated cells^15^,^16^, although high affinity binding sites have been reported in some^17^, but not all^18^ isolated membrane preparations. Thiamethoxam may be metabolized to clothianidin (CLO) or desmethyl thiamethoxam^18^, both of which are neuroactive, stimulating receptor responses^15^. In contrast, CLO is classed as a super agonist, evoking larger currents than ACh^14^. The neonicotinoids act predominantly on Kenyon cells that are the major neuronal cell type in the mushroom bodies of the bee brain^19^. These mushroom bodies are higher order brain structures mediating multisensory integration, learning and memory^20^. Given that field relevant levels^21^,^22^ of IMD and CLO have been shown to activate nAChRs in Kenyon cells^19^,^21^, it is anticipated that these compounds would influence cognitive function in bees so long as they can reach the brain at neuroactive levels following oral exposure (ie. nectar and pollen consumption). Field levels of neonicotinoids are generally accepted to be between 1–10 ppb^22^, but are not acutely lethal to bees at this level. Nevertheless, evidence on bumblebees (*Bombus terrestris audax*), indicates that neuroactive levels (~10 nM, 2.5 ppb) can reach their brain within 3 days of dietary exposure to 2.5 ppb IMD^21^. This is consistent with evidence that neonicotinoids have been shown to exert a negative impact on higher cognitive tasks in bees such as olfactory learning & memory^23^, foraging^24^,^25^, navigation^26^, queen fidelity^27^,^28^ and whole colony performance^21^,^24^,^29^, but see^30^. Neonicotinoids also disrupt the sex ratio in parasitic wasps^31^,^32^, solitary bees^33^ and possibly honeybees^34^.

The term neonicotinoid is commonly used in preference to the name of the actual compound under study in the belief that they are all essentially identical in their targets and effects. However, individual neonicotinoids, IMD, THX and CLO have been reported to exhibit distinct binding to nicotinic acetylcholine receptors^35^ and so may exert distinct effects on, and pose differential risks to bees. Indeed, as with most pharmacologically active compounds, small changes to the ligand, or the receptor binding site, may cause dramatic differences in their affinity for specific receptors. In this respect, our knowledge of the risks from neonicotinoids has been generated largely from chronic studies on IMD^21^,^25^, a cocktail of THX and CLO^27^,^33^ or THX and IMD^34^. In contrast, our knowledge on CLO, when present alone, is limited to its acute exposure effects^7^,^10^, under which conditions there is no apparent risk at field relevant levels. In the case of field trials, exposure to other known^33^,^34^,^36^ or unknown pesticides, disease or mitigating factors^30^ may occur, confounding interpretation. In this study, we compared all three EU-banned neonicotinoids, IMD, THX and CLO, for effects on bumblebees (*Bombus terrestris audax*) to determine whether they act consistently and in predictable ways, where CLO would be expected to be the most toxic, given its higher potency and THX requiring metabolism to CLO to exert an identical toxic effect.

## Results

**Figure 1:**
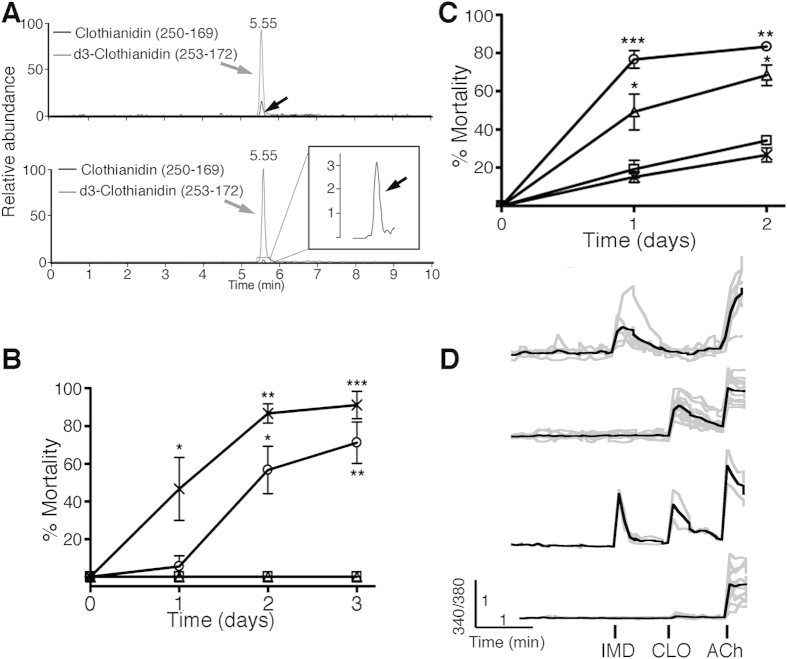
Differential neonicotinoid function on individual bees and neurons. (**A**) Representative LC-MS/MS chromatograms to indicate CLO signals detected in isolated brains derived from bees fed CLO (10 nM, upper panel) or THX (10 nM, lower panel) for 3 days. The signal from CLO is indicated with a black arrow and the spiked in d3-CLO (internal standard) is indicated with a grey arrow. CLO is eluted at around 5.55 min. (**B**) Naïve (not previously exposed to neonicotinoids) bumblebee microcolonies (30 bees) were fed untreated syrup (□) or syrup laced with 100 ppb w/v IMD (390 nM, Δ), THX (342 nM, O) or CLO (400 nM, X) for 3 days and mortality monitored on each day. The data from 3 experiments are pooled and expressed as % mortality. (**C**) Bumblebee micro-colonies (40 bees) fed untreated syrup (X) or syrup laced with field-relevant levels of neonicotinoid (10 nM): IMD (□), CLO (Δ) or THX (Ο). After 7 days (no bee deaths observed during this period), the sugar syrup was replaced with 50 ppb CLO for 1–2 days and mortality monitored on days 1 and 2. The data from 3 independent experiments are pooled and expressed as % mortality. Significance (**B**,**C**) is versus UT using two-way Anova with Bonferroni’s post-test. P-values are *≤0.05, **≤0.01 and ***≤0.001 respectively). Error bars are S.E.M (n = 3). (**D**) Calcium influx (ratiometric (340/390 nm) signal generate from a Fura-2AM probe) from individual bumblebee primary neurons in culture in response to the sequential exposure to IMD (10 nM), CLO (10 nM) and finally ACh (1 mM). The grey traces represent individual Ca^2+^ responses from single cells and the black trace represents the average response.

The first level at which differential effects of neonicotinoids could occur is by variability in their ability to reach their site of action, the bee brain. We demonstrated previously that bumblebees exposed to 10 nM (2.1 ppb w/w) IMD in their diet (sugar syrup) accumulated, within 3 days, between 4 and 10 nM within their brains^21^. Here, we demonstrate using stable isotope dilution liquid chromatography-mass spectrometry that dietary exposure to CLO (2.5 ppb) or THX (2.5 ppb), for 3 days, also delivers CLO (14.7 ± 2.0 and 3.9 ± 0.6 nM, respectively) to the brain (mean ± S.E.M, Fig. 1A), with levels of CLO in the hemolymph being 3.4 ± 0.6 (CLO fed bees) and 0.5 ± 0.1 nM (THX fed bees) respectively (Fig. 1A). Thiamethoxam levels in brains and hemolymph from THX fed bees were below the limit of quantification (Supplementary Figure S1). Therefore, as for IMD^21^, CLO and THX can deliver neuroactive levels of CLO (either as the parental form fed to bees, or via its metabolism from THX) of between 3–15 nM to the bumblebee brain.

To indicate the relative acute toxicity of IMD, THX and CLO, bees (~30) were exposed to each neonicotinoid by their inclusion in sugar syrup (100 ppb) for 1–3 days. A prior single dose-response was performed on 10 bees to indicate the level at which sub-maximal toxicity would be achieved over 3 days. Mortality to 100 ppb was determined (mean ± S.E.M, n = 3) to be 5.6 ± 5.6% (day 1), 56.7 ± 12.6% (day 2) and 71.1 ± 10.9% (day 3) for THX and 46.7 ± 16.7% (day 1), 86.7 ± 5.1% (day 2) and 91.1 ± 7.3% (day 3) for CLO (Fig. 1B), indicating a toxicity order of CLO > THX > IMD. No dead bees were observed for IMD treatment (or untreated bees), although IMD fed bees were clearly intoxicated, being immobile after overnight exposure, consistent with an anti-feedant response for IMD at this high dose, as reported previously^37^. It may be that bees are incapable of feeding or choosing to not feed.

In humans, chronic nicotine exposure leads to an upregulation of high-sensitivity (to nicotine and acetylcholine) receptors^38^ that is postulated to drive reward pathways and so contribute to the addictive actions of nicotine^39^. Similarly, the upregulation of human receptors can be induced by neonicotinoid exposure^35^, albeit at the high doses (μM) required for their receptor binding. In honeybees, neonicotinoid-seeking behavior is induced by exposure to IMD or THX, but not CLO^40^. Therefore, we investigated in bees whether a chronic exposure to field-relevant levels of neonicotinoids (2.5 ppb IMD, THX or CLO for 7 days) might increase their sensitivity to subsequent exposure to toxic levels of CLO as suggested previously^41^. As 100 ppb CLO exhibits near maximal toxicity (86.7 ± 5.1%) in naïve bees at day 2 (Fig. 1B), we assessed increased mortality using 50 ppb. We observed (40 bees) that prior exposure to field relevant levels of THX or CLO exposure caused a subsequent increased mortality to CLO (mean ± S.E.M): day 1, 76.7 ± 4.6% (THX fed) and 49.2 ± 9.4% (CLO fed), respectively; day 2, 83.3 ± 2.2% (THX fed) and 68.3 ± 5.5% (CLO fed). Naive bee mortality was lower (day 1 19.2 ± 4.6% and day 2, 34.2 ± 0.8%). Surprisingly, in contrast to the effect of THX and CLO, chronic exposure to IMD did not increase sensitivity to CLO when compared to naïve bees (day 1, 15.0 ± 2.9% and day 2, 26.7 ± 3.6%) (Fig. 1C), demonstrating that IMD does not increase sensitivity to CLO, suggesting that IMD and CLO may not activate the same receptors.

The target site for the neonicotinoids is the nicotinic acetylcholine receptors (nAChRs) commonly expressed in mushroom bodies, higher order insect brain structures that mediate multisensory integration, learning & memory^19^. Differential nAChR subtype activation by each neonicotinoid could explain the variability seen for acute toxicity, anti-feedant effects and increasing sensitivity to toxic levels of CLO (see also whole colony effects presented later). To investigate this, Kenyon cell neurons in culture were exposed sequentially to IMD (10 nM), CLO (10 nM) and then acetylcholine (1 mM) and receptor responses monitored by Ca^2+^ influx using Fura-2AM. Both IMD and CLO generate acute Ca^2+^ responses that desensitized (Ca^2+^ response returns to baseline) in the continued presence of the neonicotinoid. On the basis of a cell’s responsiveness to field-relevant levels of IMD, CLO and ACh, we determined that there are 4 distinct cell populations of Kenyon cells in our cultures, those responding to IMD only, CLO only, those responding to both IMD and CLO, or those responding to ACh only. All neurons were activated by acetylcholine (Fig. 1D) as expected for Kenyon cells. Although IMD-activated receptors are desensitized and unable to respond to subsequent activation by IMD (for example see top trace, Fig. 1D), other cells still demonstrated responses to CLO despite a prior exposure to IMD (second and third traces, Fig. 1D). In particular, in the third trace, we can see that CLO can activate receptors despite the evidence that IMD-responsive receptors have desensitized. Therefore, at a field relevant dose, IMD and CLO clearly activate distinct nAChRs and different Kenyon cell populations. Therefore, it is possible that differential receptor/Kenyon cell activation may underlie the differential effects identified here and also indicates that they may exert different risks to bumblebees and other insect pollinators.

**Figure 2:**
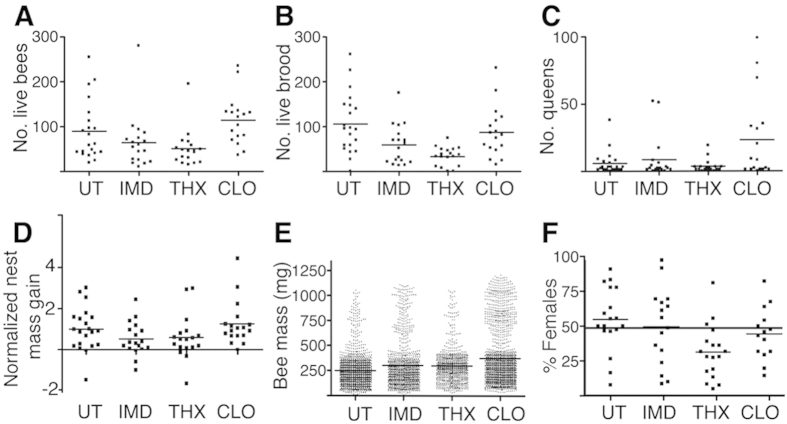
IMD and THX, but not CLO impair bumblebee colony performance at field relevant levels. Seventy-five bumblebee colonies were provided untreated sugar syrup (UT) or syrup laced with 10 nM IMD, THX or CLO and colonies were left free to forage outdoors for 5 weeks at 5 different sites across Scotland. Colonies were assessed for (**A**) the number of live bees remaining in the nest at the end of the experiment. (**B**) Viable brood remaining in the nest. (**C**) The number of queens present (the nest entrance prevents the exit of queens). (**D**) The normalized nest mass increase, relative to the control (UT) colonies of the same nest box type. (**E**) Individual bee mass for all bees is presented. (**F**) The % females in the nest were determined by counting antennal segments (12 for females and 13 for males) of 19–48 bees from each nest. All data are included to demonstrate the natural spread of performance. The average values for each treatment are indicated. Each dot represents the value from a single colony (**A**–**C**,**F**) or an individual bee (**E**).

Given these differential neonicotinoid effects, we investigated whether there is also a differential risk to bumblebee colonies in the field. Colonies were exposed to neonicotinoids (provided at 2.5 ppb in sugar syrup) over 5 weeks. Bees had full outside access after 1 day (a delay to allow transportation to site), ensuring that bees were not forced to consume the neonicotinoids as in laboratory experiments. To ensure that the relevance of our findings were not restricted to a single habitat type or period of season, we investigated the performance of 75 colonies (producing 5884 bees, 5365 brood and 727 queens) using 5 distinct sites across Scotland over 5 separate overlapping periods during the summer of 2015. Three of these sites were located within (Fife and Perth), or close to (Dundee), highly intensive arable agriculture, one site was in a mixed livestock/natural habitat (Aberfeldy) and one was in a remote, relatively pristine site in the Highlands of Scotland (Kyle of Lochalsh). To demonstrate the performance of all colonies, scatter plots of colony performance (n = 18 for each condition presented) illustrate the number of live bees, viable brood, number of queens (see supplementary Figure S3 for determination) and the normalized change in nest mass for each treatment (Fig. 2A–D).

Our estimates of colony performance are likely to be underestimates given the poor performance of the UT colonies in 2015, compared to the previous year^21^, with a reduction (±S.E.M) in mean number of live bees (138.7 ± 21.4 in 2014, versus 89.4 ± 14.4 in 2015) and mean queens produced per nest (29.2 ± 13.8 in 2014 versus 5.8 ± 1.9 in 2015). In support of this, the mean nest queen number was reported elsewhere to be 13.7 in 2011^29^ and 17.3 in 2012^42^. The poor performance of untreated colonies is likely due to the unusually cold and wet summer experienced in Scotland in 2015 that has also affected honeybee honey yield. This demonstrates the importance of weather and the risks of climate change.

Using generalized linear models (Supplementary online text) a quasi-Poisson model with log link function (live bees, brood number and number of queens), a gamma error distribution and log link function (normalized change in nest mass) or a quasi-binomial model with a logit link function (proportion females) was used. Results are summarized in Table 1.

Treatment THX is estimated to reduce the number of live bees by 38%, although the corresponding confidence interval only just excludes no effect. There is strong evidence that both IMD and THX significantly reduced number of brood cells (estimated reductions of 46% and 70% respectively). Unexpectedly, the only apparent effect on the number of queens is a significant increase under treatment CLO, relative to the control. Our finding of no evidence of a reduction in queen production contrasts with a previous study^29^. There is evidence that the normalized change in nest mass is reduced for treatment THX; estimated reduction is 10%. Finally, given the alterations to sex ratio observed in honeybees exposed to THX + IMD^34^, solitary bees exposed to THX + CLO^33^ and parasitic wasps to IMD^31^, we assessed whether bumblebee female:male ratios are also influenced by neonicotinoids. In keeping with these earlier studies, we observed a decrease in the proportion of females produced in colonies exposed to THX, but not IMD or CLO (Fig. 2F, Table 1. See also Supplemental Table S2).

Approximate F-tests on deviances indicated a significant improvement in each model when treatment was added to a model with site and period. For number of brood cells and number of queens, this result was significant at the 0.1% level; for proportion of females, at the 1% level; and for number of live bees and final nest mass, at the 5% level. QAIC (quasi-Poisson and quasi-binomial models) and AIC (gamma model) indicated that no models with any combination of interactions between site, period and treatment gave an improved fit for any of the response variables.

## Discussion

Growing evidence of the sublethal impact of field-relevant exposure to imidacloprid on honeybees indicates deficits on neuronal Kenyon cells function in the mushroom bodies^19^, which are major sites of learning and memory and multisensory integration. This neuronal dysfunction provides mechanistic insight into the evidence on honeybees for the impairment of olfactory learning and memory^23^,^43^, motor control^44^ and navigation^26^. In the case of THX, it has been demonstrated to impair olfactory memory^43^, motor control^44^ and, when exposed in combination with CLO, colony performance and queen survival^27^. In contrast to these negative impacts of THX, field studies conducted by Syngenta^30^,^45^ have reported no negative impact. However, the study and interpretations of Pilling^30^ have been contested^46^ and the other study^45^ lacked replication, with two control sites but only a single treatment site used. Therefore, further evidence is required to resolve the risk to honeybees from THX. One possible explanation for the discrepancy between laboratory and field trials is that honeybee colonies are very large and may be able to buffer the negative effects of chemical toxicity. Indeed, THX (or IMD that was found as an unexpected contaminant) has been shown to be detrimental to honeybees, causing increasing mortality over time^34^. However, the honeybee colonies appeared to compensate for the loss of workers by decreasing their production of non-working males (drones). In contrast to the mixed effects reported for IMD and THX, no effects of CLO, either alone^47^, or in combination with β–cyfluthrin^36^ were reported on honeybee colonies, suggesting that the effects reported by Sandrock^27^ may be due to a direct action of THX^48^ or its metabolic products^18^,^49^. Therefore, no clear evidence exists to implicate field-relevant exposure to CLO in whole honeybee colony losses.

For bumblebees, the evidence is more robust and consistent, with IMD being reported to impair bumblebee pollen foraging efficiency^24^,^25^ and colony impairment^21^,^24^,^29^. Similarly, exposure to THX caused bees to learn more slowly and impaired their short-term memory^50^. As for IMD, THX exposure resulted in poorer foraging and can consequentially have a negative impact on crops, with bumblebees exposed to THX providing diminished crop pollination services^51^. In contrast, in a field trial at a single site for THX-treated OSR (22 colonies), no negative impacts were reported on colony growth^45^, although no assessment of live bees remaining was possible as nests were frozen prior to assessment. In the case of clothianidin, *B. terrestris* colony growth was observed to be decreased in colonies associated with seed coated (clothiandin and β–cyfluthrin) oilseed rape, but given that a field-relevant mixture was used, the causative agent is unclear^36^.

A difficulty in assessing the field relevance of studies is that laboratory studies often do not provide a natural choice of forage and so the dose of pesticide delivered may exceed that encountered in the environment. In contrast, field studies limit exposure of bees to the field conditions under study (usually the flowering period of a crop) to minimize exposure to unknown confounding factors such as other pesticides. However, this also creates an artificial situation, with bees on site for a short duration. This has 2 problems. Firstly, a prolonged neonicotinoid exposure by its translocation to wildflowers^8^,^10^,^11^,^12^ is excluded from the study. Secondly, the expected deficit in bees is subtle, affecting their learning and memory^19^,^23^,^43^ and so their foraging ability^24^,^25^,^51^, which may not be adequately tested in a pristine habitat with (presumably) abundant and diverse wildflower availability that is lacking in a typical arable habitat.

Given the shortfalls of both laboratory and field studies in terms of field-relevance, we conducted a semi-field trial where neonicotinoids were provided as an optional supply of sugar syrup, but the bees were free to forage and needed to gather their own pollen in order to grow and raise brood. In addition, the sites selected covered a range of different habitats, from intensive arable to managed wilderness. No obvious differences between the relative effects of the neonicotinoids were observed between sites (although the sample size for between site analyses was small).

A prerequisite for the toxicity of neonicotinoids on insects is their access to nAChRs that are restricted to the brain. We demonstrate here, that all three neonicotinoids, when exposed at field relevant levels (2.5 ppb), can reach the brain in an active form at 4–15 nM (~1–4 ppb) after 3 days dietary exposure. None of the neonicotinoids are lethal to bees at field-relevant (1–10 ppb) exposure levels^22^, whereas exposure to 100 ppb causes mortality for CLO (91%) and THX (71%), but not IMD, indicating a toxicity order of CLO > THX > IMD. However, all IMD exposed (100 ppb) bees were immobile from day 1, while many CLO/THX exposed bees remained highly active. As IMD exposed bees were immobile, they do not feed^37^ and so exposure limits toxicity at high doses. Regardless, no anti-feedant effects are evident at field relevant levels and consequences from chronic exposure are possible.

Distinct consequences of particular receptor subtype activation, or as a result of differential cell stimulation may underlie the differences in acute toxicity observed and may also drive selective long-term changes following chronic exposure. Although IMD is acutely non-lethal to individual bees (at 100 ppb), it is chronically toxic to whole colonies (at 2.5 ppb), but exposure to field-relevant levels (2.5 ppb) fails to increase sensitivity to subsequent CLO exposure. THX is acutely toxic to bees and also chronically toxic to whole colonies (at 2.5 ppb). In addition, THX also alters the sex ratio in the nest and field-relevant levels increase sensitivity to subsequent exposure to high level CLO. Surprisingly, despite the acute toxicity of CLO and the ability of field-relevant levels (2.5 ppb) to increase sensitivity to high level CLO, we find no evidence for any negative effects on bumblebee colonies following a chronic exposure to CLO at field realistic levels. In fact, CLO-treated colonies produced more queens suggesting a possible beneficial impact.

To understand the potential difference between acute and chronic exposure to neonicotinoids, it may be relevant to consider the analogous situation in man, where prolonged low level exposure to nicotine occurs during smoking (and presumably vaping). Chronic nicotine administration by human smokers causes an upregulation of nicotine binding sites in the cerebral cortex^38^, a region involved in cognition, attention and memory. Indeed, acute exposure to nicotine improves attention, learning and memory. In humans, the major brain subtype is the α4β2 receptors, which are preferentially upregulated by low (nM) levels of nicotine to favor the high sensitivity stoichiometry (α4)_2_(β2)_3_ over low sensitivity (α4)_3_(β2)_2_ receptors. In addition, receptors may undergo a conformational change that results in higher affinity receptors^52^. The consequential pharmacological upregulation of function is postulated to drive reward pathways and so contribute to the addictive actions of nicotine in man^39^,^53^.

Interestingly, IMD, thiacloprid and particular metabolites have also been shown to upregulate (5–8 fold) mammalian α4β2 receptors with a potency of 500–70,000 nM^41^. In bumblebees, where neonicotinoids are more potent, we showed previously that sensitivity of nAChR was enhanced following chronic exposure to IMD^21^ supporting the case for an upregulation of receptor function. Such a mechanism is consistent with the neonicotinoid-seeking behavior reported in honeybees following exposure to IMD and THX, but not CLO^40^.

Our findings that an increased vulnerability to CLO results from exposure to field relevant levels of either CLO or THX indicates that receptor upregulation also results from chronic exposure to CLO and possibly from THX metabolites (THX does not bind to receptors). The failure of IMD to increase vulnerability to CLO appears inconsistent, unless IMD activates distinct receptors from CLO. The differential effects on individual bees and whole colonies may be the consequence of different receptor subtype function, or cell-specific roles, that may be differentially activated by individual neonicotinoids. We confirmed this potential using Kenyon cells in culture exposed sequentially to IMD, CLO and ACh. We find that most cells responded to only IMD or CLO, with few responding to both. Therefore, neonicotinoids display different cellular profiles that may drive distinct behavioral phenotypes.

The differential effect of THX on the sex bias of surviving bees in a colony is not observed for CLO (or IMD) suggesting that THX may also operate independently of its CLO metabolite. This is supported by the reported side effects of THX on the midgut of honeybees^54^ and its selective effect on grooming^44^ and may be explained by the novel metabolites produced by THX, but not CLO, such as desmethyl-THX. Desmethyl-THX has been reported to have a 28- to 3600-fold increased affinity for nAChRs over THX^15^,^18^,^55^. However, THX has also been reported to have a high binding affinity, albeit to a different site or receptor state^56^. Alternatively, it has been reported that THX and desmethyl-THX may generate formaldehyde in some species^49^ that causes protein-DNA crosslinking and chronic toxicity. Interestingly, an alteration in sex ratio by neonicotinoids has been reported previously in solitary bees^33^, honeybees^34^ and parasitic wasps^31^,^32^ and may result directly by influencing sex allocation^31^ or indirectly by possible selective loss of adult workers (female)^34^ or female pupae^32^.

In terms of differential toxicity to IMD and THX, both have been reported to impair honeybee^23^ and bumblebee^50^ learning and memory and both stimulate neonicotinoid-seeking behavior^40^. Together, these behavioral changes may underlie colony dysfunction reported here and elsewhere^21^,^24^,^29^. In contrast to IMD and THX, CLO does not appear to be detrimental to bumblebee colonies.

Our data supports the recent conclusion^17^ that there is no single conserved site for neoncitoinoid binding, but several sites with distinct binding pockets that is dependent on receptor composition. We postulate that the cells and/or specific receptor subtypes activated by CLO may not control learning and memory or drive neonicotinoid preference seeking behavior in *B. terrestris* and so does not disrupt individual bee or whole colony performance when exposed chronically at field relevant levels. It is important to bear in mind that small changes in neonicotinoid structure may have a major impact on its affinity and receptor selectivity, leading to differences in its acute and/or chronic toxicity. Equally, small changes in receptor structure, as occurs between species, presumably underlie their differential sensitivity to neonicotinoids. It is important to emphasize that the differential toxicity of IMD and THX, but not CLO, to bumblebees, may be a species-specific profile that is unique to *B. terrestris*. Therefore, we propose that the findings on individual neonicotinoids are not extrapolated to other neonicotinoids and that findings in a particular species are not extrapolated to other species.

## Methods

### Neonicotinoid feeding

Untreated sugar syrup or that laced with neonicotinoid (IMD, CLO or THX) was provided to *Bombus terrestris audax* microcolonies as the sole sugar source for the duration indicated. Microcolonies were maintained at room temperature with a natural light/dark cycle provided by sunlight via a series of large windows. Microcolonies were provided with a protein source in the form of organic pollen. Bees were sourced from 12 nests of the same age and distributed to ensure that each nest was represented in every treatment group. Bee mortality was determined via visual inspection and movement, or lack thereof, when handled. Three independent experiments were performed, from which the % mortality was derived. Prior to a comparison of acute toxicity, we performed a single dose response (10 bees for each concentration) for all 3 neonicotinoids to indicate a level that would be below the LD_100_ (dose that would kill 100% of bees) over 3 days.

### Liquid Chromatography-Tandem Mass Spectrometry (LCMS/MS) analysis

LC-MS/MS analysis was carried out using a Dionex 3000 LC system (Thermo Scientific, Hemel Hampstead, UK) linked to a Quantum Ultra Mass Spectrometer (Thermo Scientifics Hemel Hampstead, UK) with an IonMax ESI interface. A C18 column (Pursuit, 3 μm, 50 × 1 mm, ThermoFisher) with a pre-column (Pursuit 3, MetaGuard (ThermoFisher) was used to separate analytes. 5 μL of sample was injected, each sample being analysed in duplicate.

The LC was operated under gradient conditions with mobile phases of water/formic acid (99.9:0.1) (A) and acetonitrile/formic acid (99.9:0.1) (B) at a flow rate of 0.1mL/min at 30 °C. The initial mobile phase composition was 95% A which was held for 1 min, followed by a linear gradient over 5min to 95% B, held at 95% B for 1 min and then returned to 95% A over 1min. The analytical column was then equilibrated at the initial conditions for 2 min for a total run time of 10 min.

Detection was in an MRM mode, with transitions for CLO being 250->169 and d3-CLO 253->172, and THX 292-211. At the MS source, the voltage was set at 3000V, sheath gas pressure at 40, ion sweep gas pressure at 5, and auxiliary gas pressure at 8, and capillary temperature at 275 °C. The tube length offset was set at 25 and collision energy at 18V for both CLO and d3-CLO (260->213); at 20 V for THX. The scan width was 0.05 (m/z) and the resolution for Q1 and Q3 was 0.7 (FWHM). The argon pressure at Q2 was 1.2 mTorr. Data analysis was performed using XCalibur (version 2.0, Thermo Scientific) and LCQuan (version 2.5.6, Thermo Scientific). The extracted data were output to Microsoft Excel for further calculation.

### Stable isotope dilution LC-MS Analysis of IMD in brains of bees

Bees were sugar syrup with honey containing 2.5 ppb THX or CLO for 3 days. Bee brains were dissected and frozen at −80 °C prior to analysis. A total of 50 bee brains or the hemolymph from 75 bees were pooled together and three separate pooled samples were analyzed. To each sample, 12.5 μL d3-CLO (200 ng/mL, as an internal standard for quantification) and 1mL acetonitrile was added and minced on ice manually with a tissue homogeniser. The samples were then sonicated on ice for 2 × 10 sec with an ultrasonicator probe. The homogenates were centrifuged at 13,000 g for 10 min and the supernatant dried in a vacuum dryer. The samples were then reconstituted in 50 μL acetonitrile followed by addition of 950 μL 0.1% formic acid in water. A solid phase extraction using Waters HLB columns primed with 1mL acetonitrile and pre-conditioned with 0.1% formic acid in 5% acetonitrile was used to enrich analytes.

### FURA2-AM imaging of neuronal cultures

Ca^2+^ detection in primary neuronal cultures was carried out using FURA2-AM dye. Cultures were washed with phenol red free supplemented L-15 media and then incubated in the dark at 28 °C for 30 minutes in 3 μM FURA2-AM. Cells were then washed with phenol red free supplemented L-15 media at 28 °C in the dark for 15 minutes and imaged using an inverted wide-field imaging system and analysed using Volocity (PerkinElmer) software. Images were obtained under 400x magnification using excitation at 340nm/380nm with emission detection at 510nm and a capture rate of 5s. Relative Ca^2+^ signal in regions of interest was calculated using the formula:


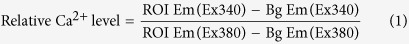


### Field experiments

Colonies were sited across 5 locations in Scotland between June and September, 2015. To avoid box effects, each colony was housed individually at and provided with sugar syrup, either untreated or laced with 2.5 ppb pesticide (IMD, THX or CLO) in the reservoir feeders situated beneath each nest. Identification markings were made near each entrance and colonies placed a minimum of 2 m apart in order to minimise orientation mistakes. All treatments were run in triplicate and treatments positioned evenly in a specific order (eg. UT-IMD-CLO-THX) and the order changed at each trial site. The 5 field sites covered a spectrum of environments from a pristine wilderness/enriched grassland habitat in Wester Ross (the Highlands), University of Dundee Botanic Garden, a livestock farming area near Aberfeldy and intensively arable landscapes in Perthshire and Fife. On the final day of each experiment, colony gates were set to “in only” for at least 12 hours before analysis. Individual nest masses were measured at the beginning and end of each experiment excluding the sugar syrup provided. Colonies were anaesthetised with CO_2_ and number of live bees determined by a combination of visual inspection and movement when handled. Bees were weighed and dispatched by decapitation before viable brood was collected and counted. Queens were identified by mass (see below) and head samples were collected from bees above and below the wax ceiling of the nest for sex ratio determination by antennal segment counting (12 for females and 13 for males).

For comparison of bee scatter profiles equal numbers of colonies are required in order to relate to the number of bees present: For CLO, one colony was discounted due to entrance being blocked in the field and the colony perished. A direct comparison of colony structure and size requires the use of identical number of colonies for each treatment. Therefore, to replace this lost colony an average CLO-treated colony was determined on the basis of having closest to the average number of queens (~25). This was determined to be colony K4.2 (20 queens). This colony’s mass spread was added to the final dataset for CLO to ensure all treatments had 18 colonies. Similarly, we had 3 additional UT colonies. Therefore, 3 colonies (F1-3.3) with a combined average number of queens (average 5.3 queens and 97 live bees; [individual values: 3, 6, 7 and 82, 43, 165 for queen and total live bees, respectively]) that most closely matched the overall average of the 21 colonies (5.8 queens and 89 live bees) were removed. The final values for the 18 UT nests are 5.9 queens and 88.2 live bees. All colonies (except the failed colony) were included in the statistical analysis. As we needed to change our nest supplier, it was necessary to account for the different nest boxes used and so for the nest mass gain, the data are normalized to the appropriate control (UT) nest boxes, with UT normalized to one.

### Estimating queen size

The minimal size (mass) of an average queen was determined by two approaches (Figure S3). Firstly, by averaging the mass of bees (53 bees) with a thorax measuring 7 mm, a criterion used to monitor queens (Dave Goulson, personal communication) but would be impractical given the number of bees (>6000) being assessed. This generated an average mass of 535 ± 90 mg (a few lighter bees could be dead, rather than anesthetized, and so slightly dehydrated). Secondly, we considered the ‘pinch point’ of bee masses (all treatments combined) to indicate where the number of bees with a particular mass reduced significantly. This was determined to be 470 mg. Therefore we applied a 535 mg cut-off to identify a queen.

### Statistical analyses

The following response variables recorded on each hive were modelled: number of live bees (nlive); number of healthy brood cells (nbrood); number of queens (nqueen); normalized change in nest mass (nestmassnorm); proportion of females (variable ‘counts’ represents counts of males and females for each colony). Because nests were provided by two different suppliers, normalized change in nest mass was calculated as the ratio (final nest mass)/(initial nest mass) divided by the ratio of (final nest mass summed across all control nests from that supplier)/(initial nest mass summed across all control nests from that supplier). For the first three response variables, a quasi-Poisson model with log link function was assumed. For normalized change in nest mass, a gamma error distribution and log link function was assumed. For proportion females, a quasibinomial model was assumed with a logit link function. For all five variables, treatments were UT, IMD, CLO, and THX. In addition, the following factors were included in the model: site (A (Aberfeldy), B (Dundee Botanic Garden), F (Fife), K (Wester Ross), P (Perth)) and period (early [June–August] or late [August–September]). For the quasibinomial model only, the estimated percentage effect of treatment is a function of site and period. As neither was found to be significant, they were excluded from the model for estimating percent reduction in proportion females under each treatment.

## Additional Information

**How to cite this article**: Moffat, C. *et al*. Neonicotinoids target distinct nicotinic acetylcholine receptors and neurons, leading to differential risks to bumblebees. *Sci. Rep.* **6**, 24764; doi: 10.1038/srep24764 (2016).

## Supplementary Material

Supplementary Information

## Figures and Tables

**Table 1:** Estimated treatment effects and tests of H_0_: no difference in mean response between treatment and control against a two-sided alternative.

Response	Treatment	Estimated effect	95% c.i.	p-value
No. oflivebees	CLO	16%	(−19%, 66%)	0.417
	IMD	−21%	(−48%, 21%)	0.276
	THX	−38%	(−60%, −2%)	0.042
No. ofbroodcells	CLO	−17%	(−42%, 18%)	0.296
	IMD	−46%	(−64%, −21%)	0.002
	THX	−70%	(−81%, −51%)	0.000
No. ofqueens	CLO	266%	( 51%, 791%)	0.005
	IMD	39%	(−51%, 292%)	0.530
	THX	−46%	(−86%, 107%)	0.359
Normalizedchange innest mass	CLO	4%	( −6%, 15%)	0.475
	IMD	−7%	(−16%, 2%)	0.137
	THX	−10%	(−19%, −1%)	0.033
PropnFemales	CLO	−17%	(−44%, 9%)	0.236
	IMD	−3%	(−32%, 25%)	0.825
	THX	−49%	(−70%, −28%)	0.001
