# Supplementary material for: Neonicotinoids target distinct nicotinic acetylcholine receptors and neurons, leading to differential risks to bumblebees
Source: Sci Rep. 2016 Apr 28;6:24764. doi: 10.1038/srep24764 (PMC4849185; doi:10.1038/srep24764)
Supplement: Supplementary Information [file srep24764-s1.docx]

**Neonicotinoids target distinct nicotinic acetylcholine receptors and neurons, leading to differential risks to bumblebees**

Christopher Moffat^1^, Stephen T. Buckland^2^, Andrew J. Samson^1^, Robin McArthur, Victor Chamosa Pino^1^, Karen A. Bollan^1^, Jeffrey T. -J. Huang^3^ & Christopher N. Connolly^1^**^*^**.

**Affiliations:**

^1^Centre for Environmental Change and Human Resilience, University of Dundee, Dundee, DD1 9SY.

^2^Centre for Research into Ecological and Environmental Modelling, University of St.Andrews, KY16 9LZ.

^3^ Biomarker and Drug Analysis Core Facility, School of Medicine, University of Dundee, Dundee, DD1 9SY.

*****Correspondence to: c.n.connolly@dundee.ac.uk


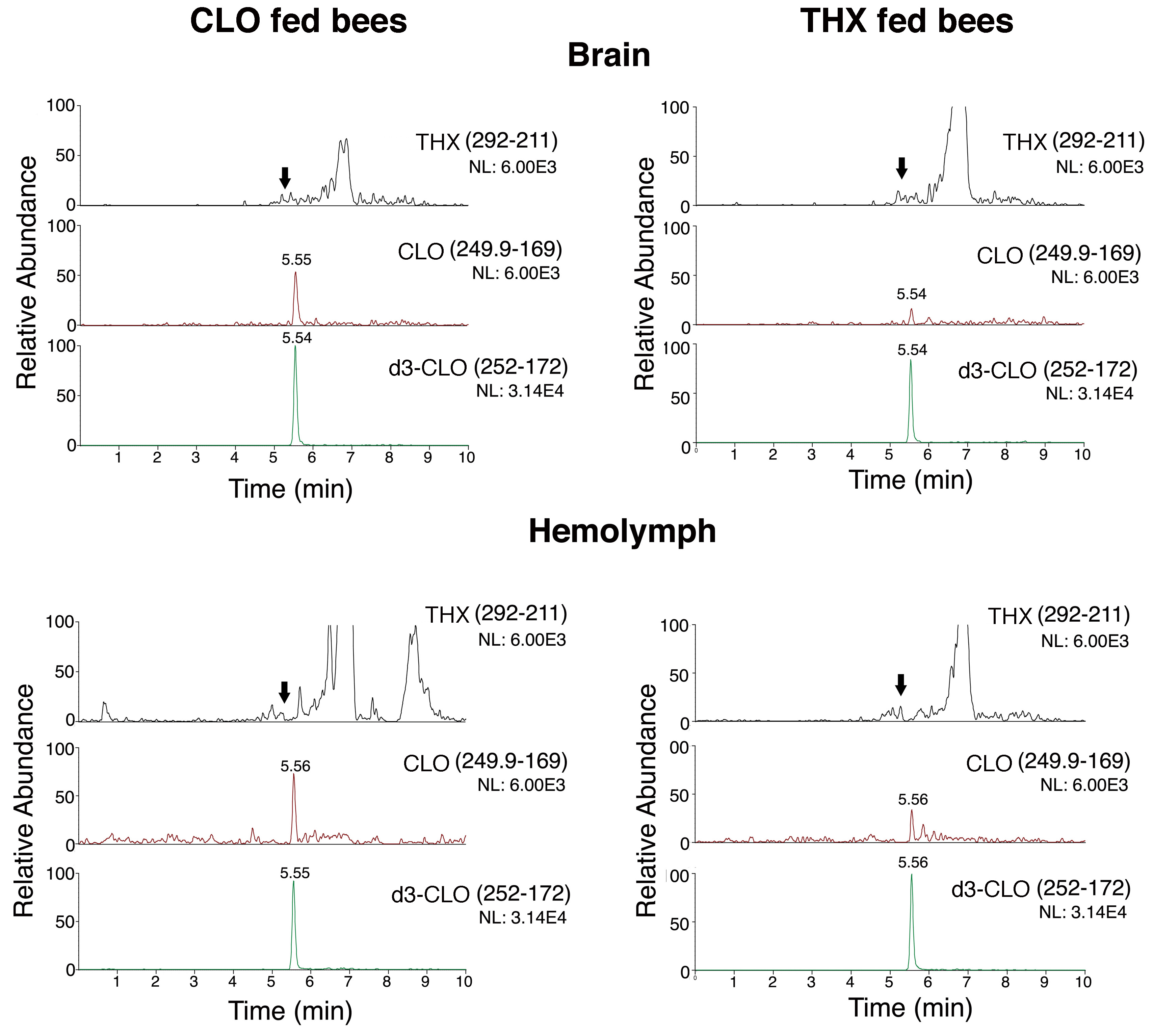


**Figure S1. Representative LC-MS/MS chromatograms of CLO and THX signals.** Bees were fed CLO or THX (2.5 ppb) for 3 days prior to removal of the brains and collection of the hemolymph. The signal from THX is shown in black, CLO is shown in red and d3-CLO (internal standard) in green. THX and CLO were eluted at 5.1min (indicated with arrows) and 5.5min, respectively. Note that the signals from THX were below limit of quantification.

|  | % Females | | | |
| --- | --- | --- | --- | --- |
|  | UT | CLO | THX | IMD |
|  | 7.7 | 14.6 | 4.8 | 8.6 |
|  | 16.7 | 20.0 | 7.5 | 10.0 (n=10) |
|  | 27.1 | 30.0 | 8.3 | 14.3 |
|  | 47.4 (n=19) | 32.4 | 13.5 | 24.2 |
|  | 47.7 | 32.5 | 17.8 | 33.3 |
|  | 48.6 | 41.2 | 19.4 | 37.1 |
|  | 51.3 | 44.2 | 28.0 | 46.2 |
|  | 51.4 | 47.2 | 28.6 | 50.0 |
|  | 51.4 | 48.9 | 28.6 | 57.5 |
|  | 56.7 | 51.4 | 32.3 | 62.9 |
|  | 57.1 | 55.6 | 32.5 | 65.9 |
|  | 60.7 | 65.7 | 37.1 | 68.7 (n=16) |
|  | 65.0 | 69.4 | 37.1 | 71.4 |
|  | 80.0 (n=20) | 84.6 | 40.0 | 71.4 |
|  | 80.0 |  | 48.3 | 94.3 |
|  | 84.4 |  | 52.6 (n=19) | 100 |
|  | 85.7 |  | 57.1 (n=14) |  |
|  | 93.5 |  | 83.3 (n=18) |  |
|  |  |  |  |  |
| Average (%) | 56.2 | 45.6 | 32.0 | 51.0 |
| STDEV | 23.4 | 19.3 | 19.9 | 28.2 |
|  |  |  |  |  |
| % colonies with  female preference | 66.7 | 35.7 | 16.7 | 53.3 |

**Table S2. Bee sexing.** Samples of bee heads were collected from nests at the end of the experiment and the number of antennal segments counted to distinguish females (12 segments) from males (13 segments). Where fewer than 25 bees were counted the numbers are shown in brackets. Figures above and below 50% are colour coded to indicate a preference for females (red) or males (blue). Finally, the % of colonies with a preference (>50%) for females is indicated.


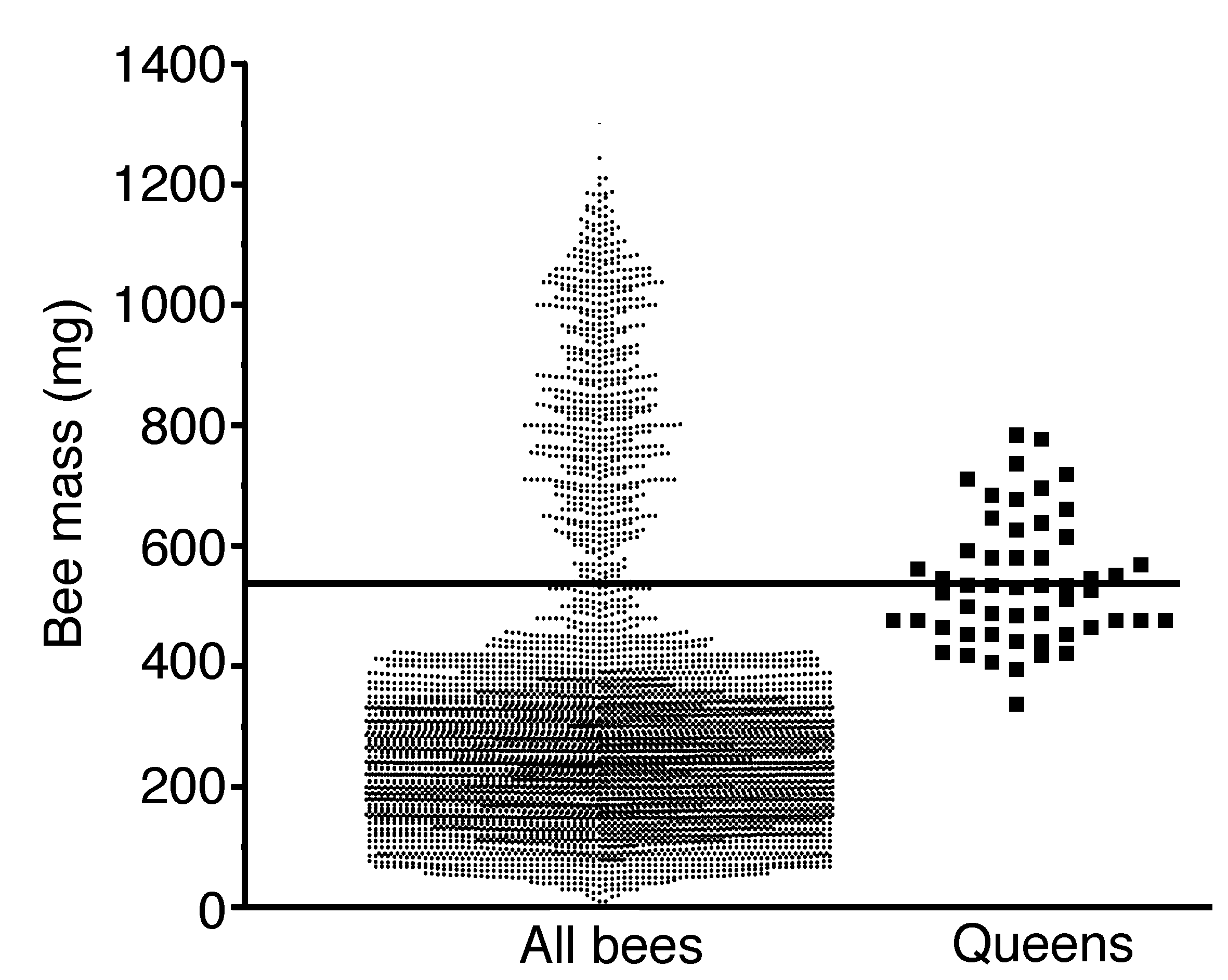


**Figure S3. Queen determination**. Queens were determined by a direct comparison to thorax width (> 7 mm) and their mass recorded (Queens) and also compared to the average colony mass distribution (All bees) to ensure that large workers were excluded.

**Supplementary data.** GLM summaries appear below:

Appendix: R output

> m1 <- glm(nlive~site+period+treat,family=quasipoisson)

> summary(m1)

Call:

glm(formula = nlive ~ site + period + treat, family = quasipoisson)

Deviance Residuals:

Min 1Q Median 3Q Max

-10.615 -3.190 -1.087 1.661 16.105

Coefficients:

Estimate Std. Error t value Pr(>|t|)

(Intercept) 4.1672 0.2373 17.562 < 2e-16 ***

siteB 0.4090 0.2600 1.573 0.12061

siteF 0.5545 0.2522 2.198 0.03150 *

siteK 0.7731 0.2848 2.715 0.00848 **

siteP -0.4745 0.4466 -1.062 0.29194

periodlate -0.1643 0.1735 -0.947 0.34698

treatCLO 0.1463 0.1793 0.816 0.41744

treatIMD -0.2318 0.2109 -1.099 0.27576

treatTHX -0.4722 0.2271 -2.079 0.04157 *

---

Signif. codes: 0 ‘***’ 0.001 ‘**’ 0.01 ‘*’ 0.05 ‘.’ 0.1 ‘ ’ 1

(Dispersion parameter for quasipoisson family taken to be 30.24137)

Null deviance: 2941.3 on 73 degrees of freedom

Residual deviance: 1813.7 on 65 degrees of freedom

AIC: NA

Number of Fisher Scoring iterations: 5

> m2 <- glm(nbrood~site+period+treat,family=quasipoisson)

> summary(m2)

Call:

glm(formula = nbrood ~ site + period + treat, family = quasipoisson)

Deviance Residuals:

Min 1Q Median 3Q Max

-12.3801 -3.9837 -0.1787 3.0980 10.0945

Coefficients:

Estimate Std. Error t value Pr(>|t|)

(Intercept) 4.33903 0.22981 18.881 < 2e-16 ***

siteB 0.19002 0.26866 0.707 0.48191

siteF 0.18218 0.26574 0.686 0.49543

siteK 0.06139 0.32856 0.187 0.85237

siteP -0.07156 0.35181 -0.203 0.83947

periodlate 0.44043 0.17725 2.485 0.01555 *

treatCLO -0.18806 0.17848 -1.054 0.29594

treatIMD -0.62497 0.19720 -3.169 0.00233 **

treatTHX -1.19552 0.24230 -4.934 5.89e-06 ***

---

Signif. codes: 0 ‘***’ 0.001 ‘**’ 0.01 ‘*’ 0.05 ‘.’ 0.1 ‘ ’ 1

(Dispersion parameter for quasipoisson family taken to be 27.26192)

Null deviance: 3129.5 on 73 degrees of freedom

Residual deviance: 1945.8 on 65 degrees of freedom

AIC: NA

Number of Fisher Scoring iterations: 5

> m3 <- glm(nqueen~site+period+treat,family=quasipoisson)

> summary(m3)

Call:

glm(formula = nqueen ~ site + period + treat, family = quasipoisson)

Deviance Residuals:

Min 1Q Median 3Q Max

-8.2616 -2.7760 -1.2352 0.7421 10.2060

Coefficients:

Estimate Std. Error t value Pr(>|t|)

(Intercept) 0.51297 0.82269 0.624 0.53512

siteB 1.30112 0.81668 1.593 0.11597

siteF 1.22419 0.81182 1.508 0.13641

siteK 0.85628 0.94174 0.909 0.36658

siteP -0.01145 1.20004 -0.010 0.99242

periodlate 0.54589 0.36197 1.508 0.13638

treatCLO 1.29805 0.44523 2.915 0.00487 **

treatIMD 0.32811 0.51927 0.632 0.52969

treatTHX -0.62479 0.67659 -0.923 0.35920

---

Signif. codes: 0 ‘***’ 0.001 ‘**’ 0.01 ‘*’ 0.05 ‘.’ 0.1 ‘ ’ 1

(Dispersion parameter for quasipoisson family taken to be 18.06707)

Null deviance: 1579.0 on 73 degrees of freedom

Residual deviance: 996.8 on 65 degrees of freedom

AIC: NA

Number of Fisher Scoring iterations: 6

> #m4 <- glm(nestmass2~site+period+treat,family=Gamma(link="log"))

> #summary(m4)

> m4 <- glm(nestmassnorm~site+period+treat,family=Gamma(link="log"))

> summary(m4)

Call:

glm(formula = nestmassnorm ~ site + period + treat, family = Gamma(link = "log"))

Deviance Residuals:

Min 1Q Median 3Q Max

-0.34448 -0.09198 -0.01992 0.08245 0.30035

Coefficients:

Estimate Std. Error t value Pr(>|t|)

(Intercept) -0.024500 0.052923 -0.463 0.6450

siteB 0.101897 0.058808 1.733 0.0879 .

siteF 0.081512 0.058042 1.404 0.1650

siteK 0.006933 0.078126 0.089 0.9296

siteP 0.153333 0.081187 1.889 0.0634 .

periodlate -0.117913 0.044311 -2.661 0.0098 **

treatCLO 0.036032 0.050197 0.718 0.4754

treatIMD -0.074802 0.049691 -1.505 0.1371

treatTHX -0.108420 0.049691 -2.182 0.0327 *

---

Signif. codes: 0 ‘***’ 0.001 ‘**’ 0.01 ‘*’ 0.05 ‘.’ 0.1 ‘ ’ 1

(Dispersion parameter for Gamma family taken to be 0.02302417)

Null deviance: 1.8750 on 73 degrees of freedom

Residual deviance: 1.4948 on 65 degrees of freedom

AIC: -65.334

Number of Fisher Scoring iterations: 5

> m5 <- glm(formula=counts ~ treat+site+period,family=quasibinomial)

> summary(m5)

Call:

glm(formula = counts ~ treat + site + period, family = quasibinomial)

Deviance Residuals:

Min 1Q Median 3Q Max

-5.7692 -1.8201 0.2525 1.6790 7.1444

Coefficients:

Estimate Std. Error t value Pr(>|t|)

(Intercept) 0.29946 0.33230 0.901 0.37122

treatCLO -0.34977 0.32752 -1.068 0.28996

treatIMD -0.06556 0.32482 -0.202 0.84076

treatTHX -1.15241 0.33771 -3.412 0.00118 **

siteB -0.34047 0.37336 -0.912 0.36558

siteF -0.28827 0.36257 -0.795 0.42981

siteP 0.01402 0.56630 0.025 0.98033

period3 0.23335 0.27989 0.834 0.40785

---

Signif. codes: 0 ‘***’ 0.001 ‘**’ 0.01 ‘*’ 0.05 ‘.’ 0.1 ‘ ’ 1

(Dispersion parameter for quasibinomial family taken to be 7.380214)

Null deviance: 603.03 on 65 degrees of freedom

Residual deviance: 481.76 on 58 degrees of freedom

AIC: NA

Number of Fisher Scoring iterations: 4
